# Functional Characterization of Domains of IPS-1 Using an Inducible Oligomerization System

**DOI:** 10.1371/journal.pone.0053578

**Published:** 2013-01-07

**Authors:** Shiori Takamatsu, Kazuhide Onoguchi, Koji Onomoto, Ryo Narita, Kiyohiro Takahasi, Fumiyoshi Ishidate, Takahiro K. Fujiwara, Mitsutoshi Yoneyama, Hiroki Kato, Takashi Fujita

**Affiliations:** 1 Laboratory of Molecular Genetics, Institute for Virus Research, Kyoto University, Kyoto, Japan; 2 Laboratory of Molecular Cell Biology, Graduate School of Biostudies, Kyoto University, Kyoto, Japan; 3 Division of Molecular Immunology, Medical Mycology Research Center, Chiba University, Chuo-ku, Chiba, Japan; 4 Institute for Innovative NanoBio Drug Discovery and Development, Graduate School of Pharmaceutical Science, Kyoto University, Kyoto, Japan; 5 Center for Meso-Bio Single-Molecule Imaging (CeMI), Institute for Integrated Cell-Material Sciences (WPI-iCeMS), Kyoto University, Kyoto, Japan; National Institute of Allergy and Infectious Diseases - Rocky Mountain Laboratories, United States of America

## Abstract

The innate immune system recognizes viral nucleic acids and stimulates cellular antiviral responses. Intracellular detection of viral RNA is mediated by the Retinoic acid inducible gene (RIG)-I Like Receptor (RLR), leading to production of type I interferon (IFN) and pro-inflammatory cytokines. Once cells are infected with a virus, RIG-I and MDA5 bind to viral RNA and undergo conformational change to transmit a signal through direct interaction with downstream CARD-containing adaptor protein, IFN-β promoter stimulator-1 (IPS-1, also referred as MAVS/VISA/Cardif). IPS-1 is composed of N-terminal Caspase Activation and Recruitment Domain (CARD), proline-rich domain, intermediate domain, and C-terminal transmembrane (TM) domain. The TM domain of IPS-1 anchors it to the mitochondrial outer membrane. It has been hypothesized that activated RLR triggers the accumulation of IPS-1, which forms oligomer as a scaffold for downstream signal proteins. However, the exact mechanisms of IPS-1-mediated signaling remain controversial. In this study, to reveal the details of IPS-1 signaling, we used an artificial oligomerization system to induce oligomerization of IPS-1 in cells. Artificial oligomerization of IPS-1 activated antiviral signaling without a viral infection. Using this system, we investigated the domain-requirement of IPS-1 for its signaling. We discovered that artificial oligomerization of IPS-1 could overcome the requirement of CARD and the TM domain. Moreover, from deletion- and point-mutant analyses, the C-terminal Tumor necrosis factor Receptor-Associated Factor (TRAF) binding motif of IPS-1 (aa. 453–460) present in the intermediate domain is critical for downstream signal transduction. Our results suggest that IPS-1 oligomerization is essential for the formation of a multiprotein signaling complex and enables downstream activation of transcription factors, Interferon Regulatory Factor 3 (IRF3) and Nuclear Factor-κB (NF-κB), leading to type I IFN and pro-inflammatory cytokine production.

## Introduction

Viruses replicating within cells produce RNA with a non-self signature, such as a double stranded (ds) and 5′-triphosphate structure, which are recognized by sensor molecules Retinoic acid Inducible Gene-I (RIG-I), Melanoma Differentiation Associated gene 5 (MDA5), and Laboratory of Genetics and Physiology 2 (LGP2), collectively known as RIG-I-Like Receptors (RLR) [1], [2], [3], [4]. RLR elicits signals to activate a set of genes including those of type I and III interferon (IFN) to initiate innate antiviral responses [5]. Several lines of evidence support a hypothesis that once RIG-I and MDA5 recognize non-self RNA, conformational changes are induced resulting in exposure of their CARD [6]. The CARD of RIG-I and MDA5 transmits a signal to another CARD-containing adaptor, Interferon Promoter Stimulator-1 (IPS-1, also known as MAVS, VISA, and Cardif), which is anchored on the outer membrane of the mitochondrion [7], [8], [9], [10]. Cells infected with a virus activate the RLR/IPS-1 signaling cascade and exhibit microscopic aggregation of IPS-1 [11]. Activation of IPS-1 is reconstituted in vitro and the formation of detergent-insoluble IPS-1 aggregate has been reported [12]. For intracellular aggregation of IPS-1, the involvement of mitofusin (MFN) 1, which is known to regulate mitochondrial fusion, has been reported [11], suggesting that IPS-1 aggregation is regulated through a complex mechanism of mitochondrial dynamics. There are several studies concerning how IPS-1 receives a signal from RLR and how it relays it downstream; however, some of the reports are not consistent with each other [10], [13], [14], [15]. IPS-1 contains three potential TRAF binding motifs (TBMs) [10]. To avoid confusion, we refer to them as TBM1 (aa. 143–147, human), TBM2 (aa. 154–159, human), and TBM3 (aa. 453–460, human). TBM1 and 2 are close to each other (5 amino acids apart) and reside within the proline-rich domain. TBM1 physically interacts with TRAF3 [16] and a single amino acid substitution (T147I) abolishes binding. Early reports demonstrated that an artificial molecule essentially consisting of CARD and TM, therefore devoid of TBMs (termed mini MAVS), is sufficient for signaling [9], [10], [13]. In particular, TM can be replaced with that of other mitochondrial proteins, suggesting the importance of its mitochondrial localization. Other reports have demonstrated that artificial oligomerization of CARD of IPS-1 in the cytosol is sufficient to activate the signal independent of the mitochondrion [14].

In the current study, we aimed to delineate the inconsistencies on the reported function of IPS-1 domains with a focus on the oligomerization of IPS-1 and analyzed the necessary part of IPS-1 for signaling.

## Results

### Forced IPS-1 Oligomerization Activates Antiviral Innate Immunity

**Figure 1:**
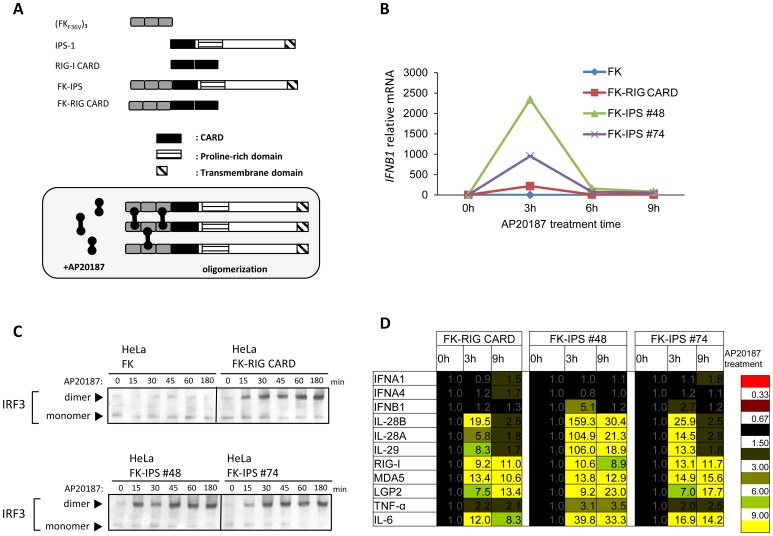
Forced IPS-1 oligomerization induced antiviral innate immune signaling. A. Schematic representation of FKBP fusion proteins and their oligomerization by a cross-linker, AP20187. B. HeLa cells stably expressing indicated FKBP fusion proteins were treated with AP20187 (10 nM) for the indicated time. Cells were harvested and analyzed for IFN-β mRNA levels by qPCR. C. HeLa cells stably expressing indicated FKBP fusion proteins were stimulated with AP20187 for 3 h and IRF-3 dimer formation was analyzed (Materials and Methods). Positions of the IRF-3 monomer and dimer are shown by arrowheads. D. Microarray analysis of mRNAs induced by oligomerized RIG-I CARD or IPS-1. Cells were stimulated with AP20187 for the indicated time. Total RNA extracted from these cells was subjected to analysis using a DNA microarray (Genopal, Mitsubishi Rayon) of interferon-stimulated genes and interferon genes. Relative mRNA levels using a control expression as 1.0 are shown. Representative data of at least two independent experiments are shown.

Previously, we found that a virus-infection resulted in the redistribution of IPS-1 to form speckle-like aggregates in cells [11]. Here, we attempted to demonstrate whether oligomerization of IPS-1 was sufficient to induce antiviral signaling. To address this question, we modified an artificial homodimerization system (ARGENT Kit, ARIAD) [17]. We used 3 tandem repeats of mutant FK 506 Binding Protein 12 (FK_F36V_), which can be cross-linked by a cell-permeable chemical AP20187 (Figure 1A). FK_F36V_ harbors an F36V mutation, which impairs binding affinity to immunosuppressive agent, FK506. AP20187 was designed specifically for binding with FK_F36V_, so that it does not influence endogenous FK binding proteins. Thus, this system specifically crosslinks a target protein without the unwanted side effects. We made constructs to artificially oligomerize CARD of RIG-I in cells (FK-RIG CARD) [18] and IPS-1 (FK-IPS) (Figure 1A). HeLa cells stably expressing 3xFK_F36V_ (FK) and its fusion proteins were treated with AP20187 and IFN-β mRNA levels were quantified. AP20187 induced oligomerization of fusion proteins (Native PAGE, data not shown). Oligomerization of FK_F36V_ did not induce IFN-β mRNA; however, FK-RIG CARD exhibited a rapid induction of IFN-β mRNA (Figure 1B, [18]). Two independent HeLa clones expressing FK-IPS activated the IFN-β gene upon AP20187 treatment, both of which expressed the fusion protein localized to mitochondria (data not shown). Furthermore, AP20187 treatment induced speckle-like distribution of FK-IPS in cells (data not shown). It is important to note that unlike transient overexpression of IPS-1 in cell lines, which constitutively activates the IFN-β gene; stable cells did not exhibit constitutive IFN-β expression (Figure 1B). To confirm that this induction was accompanied by activation of IRF-3, its dimer formation was examined by native PAGE (Figure 1C). Consistent with IFN-β mRNA levels, cells expressing FK-RIG and FK-IPS, but not FK exhibited rapid IRF-3 dimer formation after exposure to AP20187.

To further confirm the impact of antiviral signaling by this artificial system, we examined expression profiles of interferon stimulated genes by a DNA microarray of 237 immune-related genes. 109 genes were transiently induced by IPS-1 oligomer (data not shown). Representatives 11 genes, which are known to be induced after a viral infection, are displayed in Figure 1D. Results show that a simple oligomerization of FK-RIG CARD or FK-IPS mimics the signaling induced by a viral infection (Figure 1D). In contrast, only CARD of IPS-1 failed to induce any interferon or cytokine gene expression in response to oligomerization (Figure S1). From this, it would appear that the up-regulations of these genes are not due to non-specific response induced by oligomerized CARD-containing protein.

### CARD of IPS-1 is Dispensable for Oligomerization-induced Signaling

**Figure 2:**
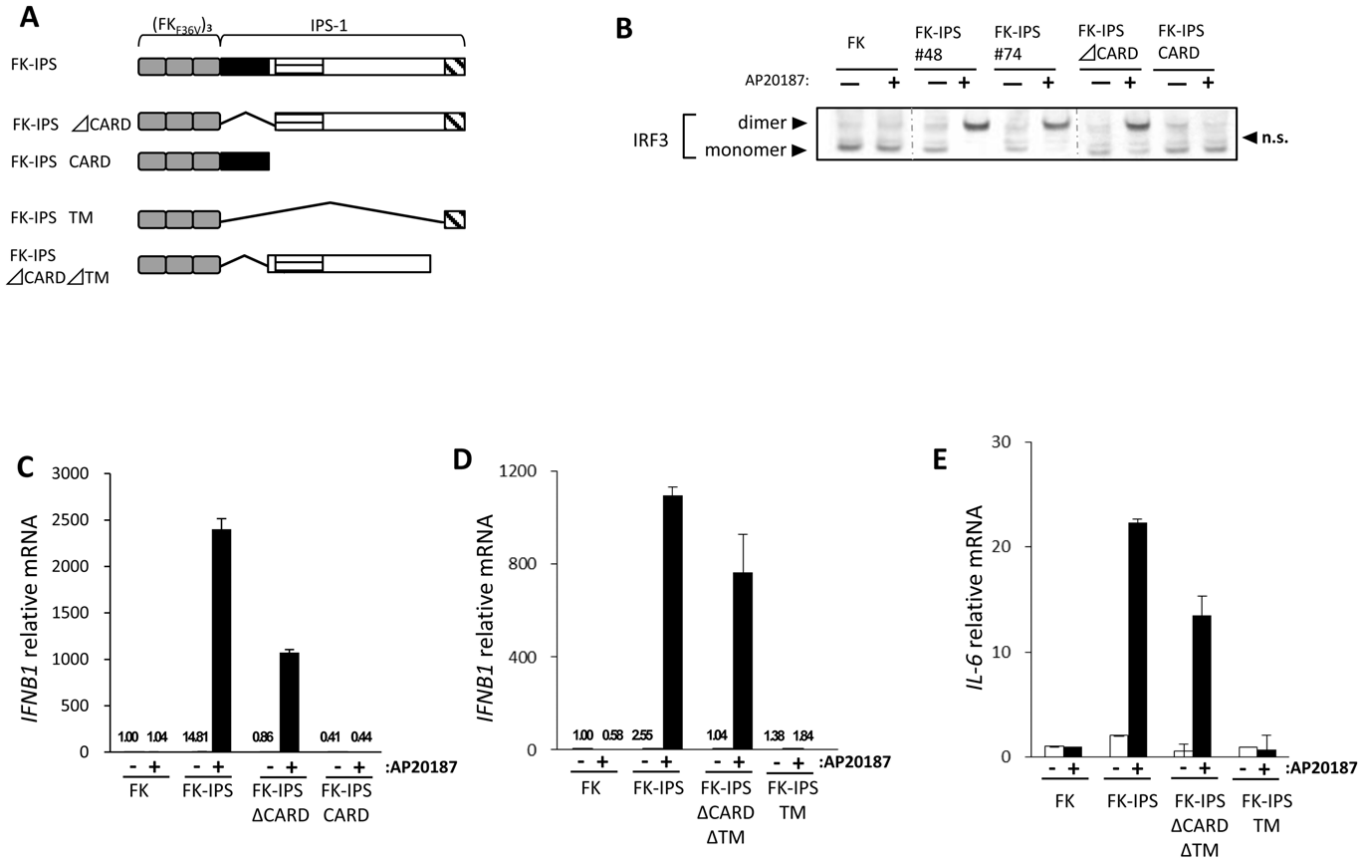
CARD of IPS-1 is dispensable for oligomerization-induced signaling. A. Schematic representation of FK-IPS deletion mutants. B. HeLa cells stably expressing indicated FK-IPS fusion were mock treated or treated with AP20187 for 3 h. Cell lysates were analyzed for IRF-3 dimer formation as in Figure 1C. n.s.: non-specific band. C–E. Indicated HeLa cells stably expressing FK-IPS constructs were mock treated or treated with AP20187 for 3 h. Cellular RNA were extracted and analyzed for IFN-β (C, D) or Il-6 (E) mRNA by qPCR. Representative data of at least two independent experiments are shown. Error bars: standard error of triplicated samples.

It has been hypothesized that upon activation of RIG-I, its CARD is exposed by conformational changes and relays signaling to IPS-1 through CARD-CARD interactions [8], [9], [10], [19]. CARD of IPS-1 is essential for signaling when IPS-1 is transiently over-expressed [7], [8]. We examined if CARD of IPS-1 was essential in FK-IPS-mediated signaling. We constructed FK-IPS mutants: FK-IPSΔCARD, CARD deletion; FK-IPSCARD, FK fused to CARD; FK-IPSTM, FK fused to TM, and FK-IPSΔCARDΔTM, FK-IPS without CARD and TM, as summarized in Figure 2A. Stable cells expressing FK-IPSΔCARD showed little basal activation of IRF-3; upon treatment with AP20187, strong activation of IRF-3 was observed similar to that by FK-IPS (Figure 2B). However, FK-IPS CARD did not activate IRF-3 even after oligomerization (Figure 2B). Similarly, FK-IPSΔCARD, but not FK-IPSCARD did not induce IFN-β mRNA upon oligomerization (Figure 2C). Oligomerization of FK on the mitochondrion (FK-IPSTM) is not sufficient to activate IFN-β and Interleukin(IL)-6 genes (Figure 2D, 2E). Interestingly, FK-IPSΔCARDΔTM, which is localized in the cytoplasm (Figure S2) due to TM deletions, activated IFN-β and IL-6 genes and formed speckle-like aggregates upon oligomerization (Figure 2D, 2E, and Figure S2). These results suggest that cytoplasmic oligomerization of an IPS-1 fragment (aa. 90–507), which includes TBM1–3, is sufficient for signaling and mitochondrial localization is dispensable if forcibly oligomerized.

### Domain Delimitation of IPS-1 for IRF3 and NF-κB Activation

**Figure 3:**
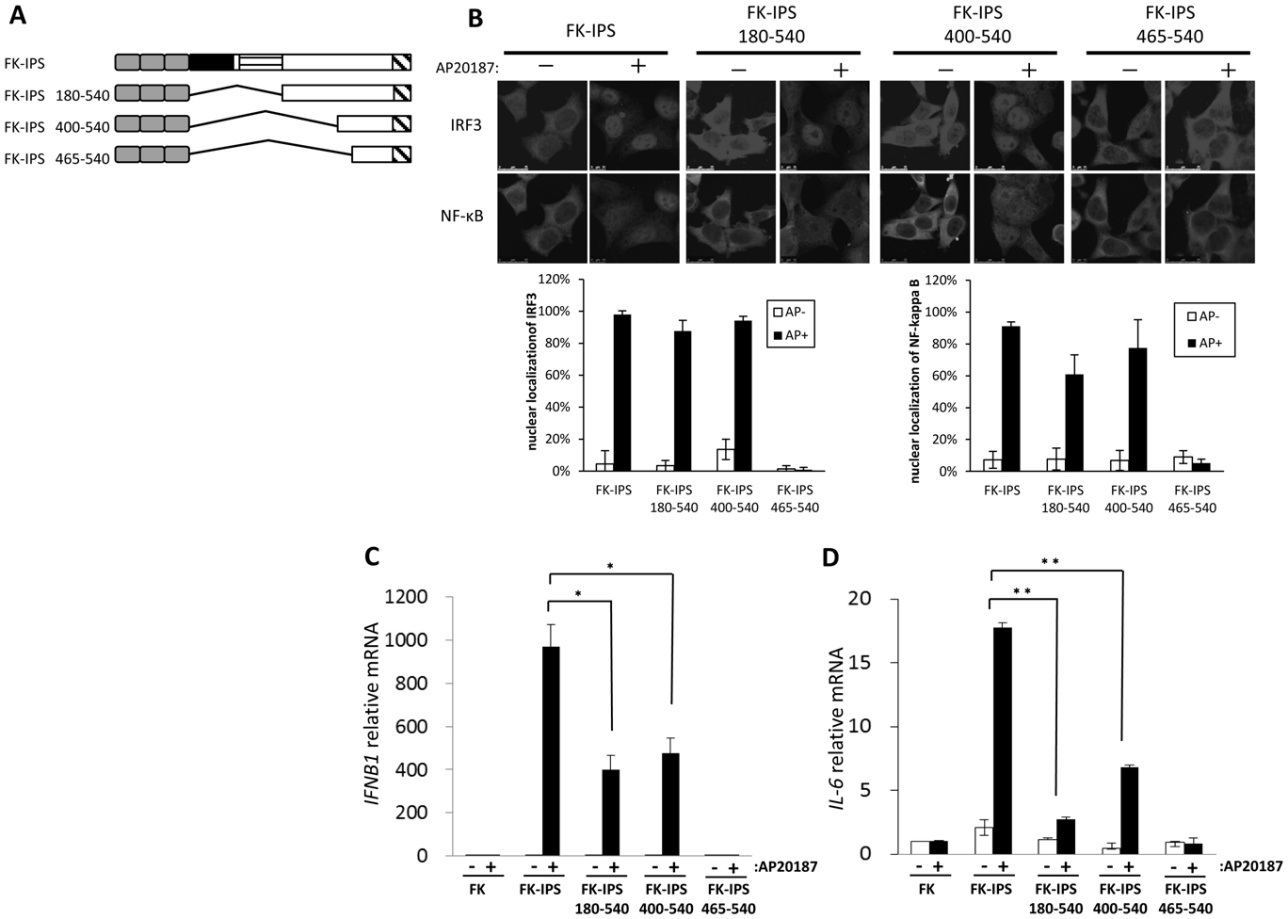
Delimitation of critical domain in IPS-1 for IRF3 and NF-κB activation. A. Schematic representation of FK-IPS deletion mutants. B. HeLa cells stably expressing indicated FK-IPS deletion mutants were mock treated or treated with AP20187 for 3 h. Cell were fixed and stained for IRF-3 and NF-κB p65, respectively. Fluorescent microscopic images of IRF3 and NF-κB staining are shown (top). The percentage of cells with nuclear IRF-3 or NF-κB was determined by counting 100 cells (bottom). C, D. Cellular RNA was extracted and analyzed for IFN-β (C) or IL-6 (D) mRNA by qPCR. Representative data of at least two independent experiments are shown. Error bars: standard error of triplicated samples. Statistical analyses were conducted with an unpaired t test, with values of p<0.05 considered statistically significant. *p<0.05, **p<0.005.

To delimit the region of IPS-1 necessary to trigger signaling upon oligomerization, we made a series of deletion mutants as shown in Figure 3A. Stable clones of HeLa cells expressing these mutants were mock treated or treated with AP20187 and nuclear translocation of IRF-3 and NF-κB was determined by immunostaining (Figure 3B). Deletion of the proline-rich region (180–540) showed little effect; however, further deletion of residues 400 to 464 abolished activation of both IRF-3 and NF-κB, indicating that these residues are essential to signal. Quantitative analysis of IFN-β and IL-6 gene expression revealed a significant attenuation of signaling by the deletion of aa. 1–179 (Figure 3C, 3D), suggesting the involvement of TBM1 and 2. This requirement of TBM1–2 is more prominent for IL-6 gene expression. Importantly, further deletion of aa. 400 to 464 (FK_F36V_-IPS 465–540), including TBM3, resulted in the complete loss of signaling activity.

We also wondered whether MFN1 contributes to IPS-1 oligomerization because we previously reported that mitochondrial protein MFN1 promotes mitochondrial fusion and increases signaling of IPS-1 [11]. We carried out a reporter assay with this oligomerization system in MFN1−/− MEFs. MFN1−/− MEFs showed comparable level of IFN-promoter activity to WT MEF cells (Figure S3), suggesting that MFN1 is dispensable for signaling induced by forced oligomerization of IPS-1.

### Essential Role of TBM3 in Signaling

**Figure 4:**
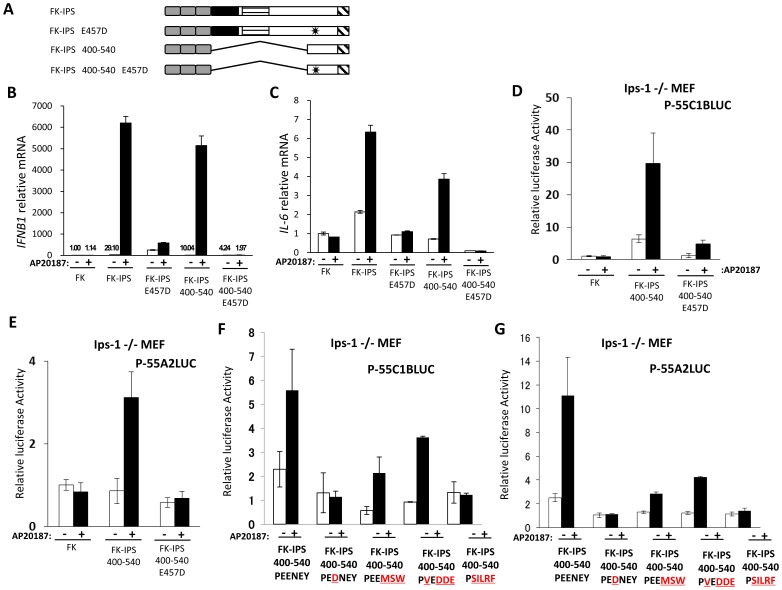
Essential role of TBM3 in signaling. A. Schematic representation of FK-IPS fusion proteins. Asterisks represent the point mutation. B, C. HeLa cells stably expressing indicated FK-IPS mutants were mock treated or treated with AP20187 for 3 h. Cellular RNA were extracted and analyzed for IFN-β (B) or IL-6 (C) mRNA by qPCR. D–G. IPS-1−/− MEFs were transiently transfected with the luciferase reporter plasmid, p-55C1BLuc (for IRF, D, F) or p-55A2Luc (for NF-κB, E, G), together with indicated FK-IPS-1 fusion constructs. For TBM3 mutants, substituted amino acids are shown as red letters (F, G). Cells were treated with or without AP20187 for 6 h. Relative luciferase activities were determined as described in the Materials and Methods. A representative result of at least two independent experiments is shown. Error bars: standard error of triplicated samples.

To further characterize functional residues within aa. 400–540, we substituted a single amino acid within TBM3 (PEENEY to PEDNEY: E457D) [10] to explore its significance (Figure 4A). E457D substitution abolished gene activation of IFN-β and IL-6 with full-length or 400–540 FK_F36V_ fusion constructs in stable HeLa cells (Figure 4B, 4C). We confirmed that IRF and NF-κB were activated by oligomerization of IPS-1 400–540 in a TBM3-dependent manner (Figure 4D, 4E). We further mutagenized TBM3 to resemble TBM of Toll/IL-1 receptor domain-containing adaptor inducing IFN-β (TRIF) (PEEMSW) or IL-1 receptor-associated kinase (IRAK)-M (PVEDDE). As a negative control, the motif was replaced to that of Myeloid Differentiation factor 88 (MyD88) (PSILRF), which does not bind directly to the TRAF molecule [20]. Interestingly, substitution of TBM3 with TBM of TRIF or IRAK-M restored the induction of IRF3 and NF-κB, albeit with lower efficiency (Figure 4F, 4G). As expected, the control motif of MyD88 failed to exhibit signaling. Furthermore, we constructed FK-IPS 400–508, which retains TBM3 but lacks the TM. This short fragment of IPS-1 also activated IRF-responsive promoter upon oligomerization(Figure S4). This result further supports the hypothesis that oligomerization of TBM3 is essential in IPS-1 mediated signaling.

### Viral Infection Induces Molecular Oligomer of IPS-1

**Figure 5:**
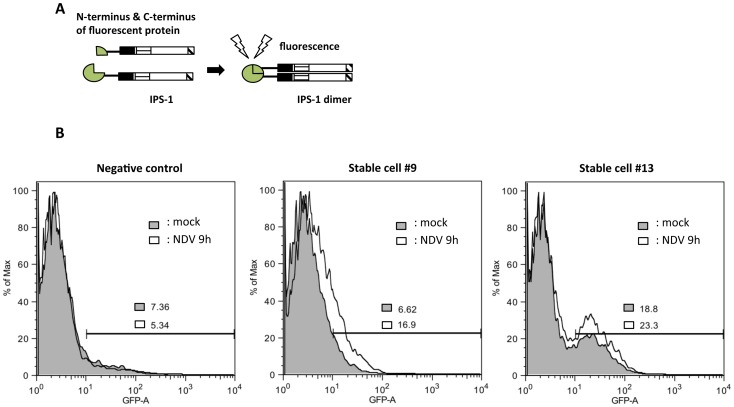
Viral infection induces the molecular oligomer of IPS-1. A. Schematic representation of dimers detection by mKG-tagged IPS-1. B. Flow cytometry plots of control 293T cells and 2 clones stably expressing mKG-tagged IPS-1, #9 and #13. The cells were mock treated or infected with NDV for 9 h. Cells exhibiting fluorescent intensity >10^1^ were quantified and expressed as % of total cell number.

The above results show that forced oligomerization of IPS-1 results in the activation of a signaling cascade. We investigated if a viral infection induced oligomerization of IPS-1 using fusion proteins of complementary fragments of a fluorescent reporter protein (monomeric Kusabira-Green, mKG) [21]. Two split inactive mKG fragments fused to IPS-1, respectively, were expressed in cells. Fluorescence is expected to be detectable when these IPS-1 fusions containing complementary mKG fragment came into close vicinity (Figure 5A). 293T cells, which stably expressed mKG-fusion IPS-1, were infected with Newcastle disease virus (NDV) for 9h and then subjected to Fluorescence-Activated Cell Sorting (FACS) analysis for the detection of fluorescence. We observed enhanced fluorescence in NDV-infected cells (Figure 5B), suggesting that viral infections induce oligomer formation of IPS-1.

## Discussion

Signaling initiated by cytoplasmic viral RNA sensors involves a unique adaptor, IPS-1, which is specifically expressed on the outer membrane of the mitochondrion. IPS-1 is a problematic protein, since transient overexpression results in constitutive signaling, whereas endogenous IPS-1 is tightly regulated by post-translational mechanisms [22], [23]. Here, we established a system to analyze the regulation of IPS-1 by its oligomerization. We obtained stable cell lines expressing FK-IPS fusion, which could be activated by a crosslinker. Upon oligomerization, IPS-1 rapidly elicited signaling leading to the activation of target genes including that of IFN-β, suggesting that IPS-1 aggregation is essential and precedes possible covalent modifications such as phosphorylation and ubiquitination [24], [25].

Our deletion analysis of FK-IPS-1 revealed that the TRAF binding motif is essential while CARD is dispensable for signaling. The initial report by Chen’s group reported that CARD tethered to mitochondria-targeted TM (termed mini MAVS) is sufficient to transduce signaling by its transient overexpression [9], [13]. They expressed mini-MAVS in cells expressing endogenous IPS-1. However, when mini-MAVS was expressed in IPS-1−/− cells, no signal was transduced (Figure S5, [26]). And recently Chen’s group also reported that depletion of endogenous IPS-1 by RNAi abrogated interferon induction by mini-MAVS [12]. This can be interpreted as transient overexpression of CARD in the vicinity of mitochondria resulting in the aggregation of endogenous IPS-1. In contrast, FK-IPS 400–450, which lacks CARD, is regulated by oligomerization in IPS-1−/− MEFs (Figure 4D, 4E). Another group showed that cytoplasmic oligomerization of CARD is sufficient to activate signaling using FK fusion [14]. This result is clearly inconsistent with ours (Figure 2B, 2C). They used wild type FKBP12 and dimerizer chemical AP1510, which retains its binding affinity to endogenous FKBP proteins. One of the FKBPs, FKBP38 (also termed FKBP8) is known to associate with the mitochondrial outer membrane [27]. Therefore, this primordial oligomerization system may oligomerize the target proteins (this case CARD) in association with mitochondria. We used an improved FKBP system (ARGENT Kit, ARIAD), which avoids this potential problem. On the other hand, FK-IPS ΔCARDΔTM, which contains TBMs, can activate signaling upon oligomerization (Figure 2). This result highlights the fact that cytoplasmic oligomerization of TBMs is sufficient for signaling.

There are three potential TBMs within IPS-1 [10]. Our result showing that FK-IPS 400–540 exhibited signaling in an oligomerization-dependent manner (Figures 3 and 4) suggest that oligomerization of TBM 3 alone is sufficient for signaling. TBM3, initially identified as TRAF6 binding site [10], can also recruit TRAF3 [28]. This is consistent with studies using TRAF3 and TRAF6 knockout cells [29], [30]. TBM1, 2, and 3 likely contribute to the signaling mediated by IPS-1, presumably in a cooperative fashion and result in differential activation of target genes. For example, TBM1 and 2 are dispensable for the IFN-β gene, but IL-6 gene requires all TBM1, 2, and 3 for full activation (Figure 3C, 3D). A recent report has shown that CARD containing protein CARD9 is preferentially required for proinflammatory cytokine induction downstream of RIG-I signaling [31]. To explore the involvement of CARD9 in IPS-1 mediated signaling, we knocked down CARD9 in a stable HeLa clone expressing FK-IPS and examined its effect on the activation of IFN-β and IL-6 genes (Figure S7). Although IFN-β gene induction by oligomerization was little affected by reducing CARD9, IL-6 gene activation was significantly attenuated. Considering the result that IL-6 gene activation is more dependent on TBM1/2 (Figure 3C, 3D), it is tempting to speculate that TBM1/2 preferentially promote NF-κB activation, whereas TBM3 has a primary role of IRF-3/7 activation.Our results support a model that CARD of IPS-1 receives signaling from RLR via CARD-CARD interaction to initiate oligomerization through mitochondrial dynamism; however, CARD of IPS-1 alone is not sufficient to trigger downstream signaling. On the other hand, TBMs are essential for further signaling by the recruitment of TRAF3 and 6, which is initiated by molecular oligomerization. Consistent with this model, we observed that artificial oligomerization of IPS-1 induced recruitment of TRAF6 into the NP-40-insoluble fraction (Figure S6). Thus, IPS-1 receives and transmits signaling through the functions of CARD and the TRAF motif, respectively.

## Materials and Methods

### Plasmid Constructs

p-55C1BLuc, p-55A2Luc, p-125Luc, pRLtk, pEF-Bos-FLAG-RIG-I CARD and pEF-Bos-FLAG-IPS-1 plasmids have been described [11], [32]. Expression plasmids of FKBP36v (oligomerization peptide), pC4M-Fv2E, and pC4Fv1E were obtained from ARIAD (ARGENT Regulated Homodimerization kit). We re-constructed the vector, pC4Fv3E, which contains 3 tandem repeats of FKBP36v [18]. To construct IPS-1 fused three tandem FKBP, we amplified the IPS-1 sequence by PCR and inserted it into the SpeI site of pC4Fv3E. Site-directed FK-fused IPS-1 mutants (FK-IPS E457D, FK-IPS 400–540 E457D) were constructed using a KOD-Plus mutagenesis kit (TOYOBO, Japan). Nucleotide sequences for these constructs were confirmed with the BigDye DNA sequencing kit (Applied Biosystems). Expression vectors encording Flag-MAVS and Flag-mini-MAVS were obtained from Dr. Zhijian J. Chen.

### Cell, DNA Transfection, and Preparation of Cell Extracts

HeLa, 293T cells [32], [33] and Mouse embryonic fibroblasts (MEFs) [5], [34] were maintained in Dulbecco’s Modified Eagle’s Medium with 10% fetal bovine serum and penicillin-streptomycin. MEFs deficient for IPS-1 were obtained from Dr. S. Akira (Osaka University). MEFs deficient in MFN1 were obtained from Dr. David Chan (Caltech). HeLa, 293T cells, and MEFs were transfected with FuGENE 6 (Roche Applied Science). Stable transformants of HeLa cells were established by transfection of linearized plasmids, encoding the FKBP construct and Puromycin resistance gene, respectively, and cells were selected by Puromycin (5 µg/ml). For preparation of cell extracts, cells were lysed with lysis buffer (50 mM Tris-HCl pH 7.5, 150 mM NaCl, 1 mM EDTA, 1% Nonidet P-40, 0.1 mg/ml leupeptin, 1 mM phenylmethylsulfonyl fluoride, and 1 mM sodium orthovanadate) and were centrifuged at 20400×g for 10 min. The supernatant was used for immunoblotting.

### Viral Unfection

Cells were treated with culture medium or infected with NDV at a MOI of 1 in serum-free and antibiotic-free medium. After adsorption for 1 h at 37°C, the medium was changed and infection was continued for 9 h in the precence of serum-containing medium.

### Reporter Assay

MEFs were transfected with firefly luciferase reporter (either p-125 Luc, p-55C1BLuc or p-55A2Luc [32]) pRLtk (renilla luciferase internal control) and effector expression plasmids. Cells were split into three aliquots and were stimulated with chemical dimerizer AP20187 (AP, 10 ng/ml in ethanol) or ethanol. The luciferase assay was performed with a Dual-Luciferase reporter assay system (Promega). Luciferase activity was normalized using *Renilla* luciferase activity (pRLtk).

### Quantitative Real Time PCR and Microarray Analysis

Total RNA was prepared with TRIZOL reagent (Invitrogen) and treated with DNase I (Roche Diagnostics). A High-Capacity cDNA Reverse Transcription Kit (Applied Biosystems) was used for cDNA synthesis and mRNA levels were monitored with the Step One plus Real Time PCR system and TaqMan Fast Universal PCR Master Mix (Applied Biosystems). TaqMan primer-probes for human IFNB1, IL-6, IFNA8, and 18 s rRNA were purchased from Applied Biosystems. RNA copy numbers were normalized to that of an internal 18 s rRNA. In the microarray analysis, we used the Genopal microarray system according to the manufacturer’s instructions (Mitsubishi Rayon). Biotin-labeled RNA was prepared with a MessageAmp II-Biotin Enhanced kit (Ambion).

### Immunoblotting and Antibodies

The polyclonal antibody used to detect human IRF-3 in native PAGE and anti-human IRF-3 polyclonal antibodies for immunostaining were described previously [35]. Other antibodies were obtained from the following sources: Anti-human NF-**κ**B antibody (sc-109), anti-human TRAF6 (sc-8409), and anti-human MFN1 (sc-50330) from Santa Cruz Biotechnology, anti-HA-Tag (6E2) from Cell Signaling, and anti-human Actin (A-1978) from Sigma.

### Immunofluorescence Microscopy

For immunofluorescence analysis, cells were fixed with 4% paraformaldehyde for 10 min, permeabilized with acetone: methanol (1∶1), and blocked with 5 mg/ml of BSA in PBST (0.04% Teen20 in PBS) for 1hour. Cells were incubated with relevant primary antibodies overnight at 4°C, then incubated with Alexa Fluor-conjugated secondary antibodies (Invitrogen). To label mitochondria, cells were incubated for 30 min at 37°C with MitoTracker Red CMXRos according to the manufacturer’s instructions (Molecular Probes). Fluorescence images were obtained by Leica Microsystems AF6500 (Leica).

### RNA Interference

The siRNA negative control, targeting TRAF3 and TRAF6 were purchased from Bonac Corporation. The target sequences were: (GCUCAUGGAUGCUGUGCAUdTdT) and (GGAGAAACCUGUUGUGAUUdTdT) for TRAF3 and 6, respectively. Each siRNA was transfected with Lipofectamine 2000 (Invitrogen) according to the manufacturer’s instructions. At 48 h post-transfection, cells were harvested, and then subjected to Real Time PCR.

### FACS

To examine oligomerization of IPS-1 in cells, we performed bimolecular fluorescence complementation (BiFC) assays using a CoralHue Fluo-Chase kit (Amalgam).

293T cells expressing this construct were washed and harvested with PBS, then subjected to FACS analysis using FACSCanto II (BD Bioscience).

## Supporting Information

Figure S1 **Microarray analysis of mRNAs induced by oligomerized IPS-1 CARD or IPS-1.** HeLa cells stably expressing FK-IPS or FK-IPS CARD were stimulated with AP20187 for the indicated time. Total RNA extracted from these cells was subjected to analysis using a DNA microarray (Genopal, Mitsubishi Rayon) of interferon-stimulated genes and interferon genes. Relative mRNA levels using a control expression as 1.0 are shown.
(PDF)

Figure S2 **FK-IPS** Δ**CARD**Δ**TM forms speckle like aggregates in the cytoplasm.** HeLa cells stably expressing FK-IPS ΔCARDΔTM were mock treated or treated with AP20187 for 3 h and stained with mitoTracker (mitochondria) and anti-HA antibody. Fluorescent microscopic images of FK-IPSΔCARDΔTM and mitochondria are shown.
(PDF)

Figure S3 **MFN1 is dispensable for signaling induced by forced oligomerization of IPS-1.** MEFs of MFN1−/− or +/+ were transiently transfected with p-125Luc (reporter for IFN-β promoter activity) together with the indicated FK-IPS fusion constructs. Cells were treated with or without AP20187 for 6 h. Relative luciferase activities were determined as described in Materials and Methods. A representative result of at least two independent experiments is shown. Error bars indicate standard error of triplicate samples.
(PDF)

Figure S4 **FK-IPS 400–508 can activate IRF-responsive promoter upon oligomerization.** HEK 293T cells were transiently transfected with p-55C1BLuc together with the FK or FK-IPS 400–540 constructs. Cells were treated with or without AP20187 for 6 h. Relative luciferase activities were determined as described in Materials and Methods. A representative result of at least two independent experiments is shown. Error bars indicate standard error of triplicate samples.
(PDF)

Figure S5 **IPS-1**Δ**100–500 (mini-MAVS) failed to activate signaling in the absence of endogenous IPS-1.** IPS-1−/− or +/+ MEFs were transiently transfected with luciferase reporter plasmid, p-55C1BLuc together with IPS-1(MAVS), IPS-1Δ100–500 (mini-MAVS), or control vector. Relative luciferase activities were determined as described in Materials and Methods. A representative result of at least two independent experiments is shown. Error bars indicate standard error of triplicate samples.
(PDF)

Figure S6 **Recruitment of TRAF6 into NP-40 insoluble fraction upon oligomerization of IPS-1. A.** Scheme for isolation of soluble and insoluble fractions by differential centrifugation. **B and C.** Immunoblot analysis of soluble/insoluble fractions separated by differential centrifugation. FK-IPS ΔCARD stable cells were cultured for 3 h in the absence or presence of AP. Cell lysates were separated by differential centrifugation. FK-IPS ΔCARD and endogenous MFN1, TRAF6, and actin were detected by immunoblotting.
(PDF)

Figure S7 **Involvement of CARD9 in NF-κB dependent pathway. A.** HeLa FK-IPS#48 cells were transfected with N.C. siRNA or CARD9 targeted siRNA for 48 h, and the knockdown of CARD9 was analyzed by RT-PCR. **B, C and D.** HeLa FK-IPS#48 cells were transfected with N.C. siRNA or CARD9 targeted siRNA for 48 h, then mock treated or treated with AP20187 for 3 h. Cellular RNA were extracted and analyzed for IFN-β (B), Il-6 (C) or Il-1β (D) mRNA by qPCR. Representative data of at least two independent experiments are shown. Error bars: standard error of triplicated samples. Statistical analyses were conducted with an unpaired t test, with values of p<0.05 considered statistically significant. *p<0.05.
(PDF)
