# Supplementary material for: Functional Characterization of Domains of IPS-1 Using an Inducible Oligomerization System
Source: PLoS One. 2013 Jan 7;8(1):e53578. doi: 10.1371/journal.pone.0053578 (PMC3538592; doi:10.1371/journal.pone.0053578)
Supplement: Figure S1 — Microarray analysis of mRNAs induced by oligomerized IPS-1 CARD or IPS-1. HeLa cells stably expressing FK-IPS or FK-IPS CARD were stimulated with AP20187 for the indicated time. Total RNA extracted from these cells was subjected to analysis using a DNA microarray (Genopal, Mitsubishi Rayon) of interferon-stimulated genes and interferon genes. Relative mRNA levels using a control expression as 1.0 are shown. (PDF) [file pone.0053578.s001.pdf]

Supporting Information

Supplementary Figure 1

	FK-IPS CARD			FK-IPS #48		
	0 h	3h	9h	0h	3h	9h
IFNA1	1.0	0.7	1.2	1.0	0.7	0.6
IFNA4	1.0	0.7	1.5	1.0	0.7	0.6
IFNB1	1.0	0.6	1.4	1.0	3.0	0.7
IL-28B	1.0	0.8	1.4	1.0	71.4	8.1
IL-28A	1.0	0.6	1.3	1.0	39.2	5.1
IL-29	1.0	0.6	1.1	1.0	44.1	4.4
RIG-I	1.0	0.8	1.1	1.0	10.5	9.5
MDA5	1.0	0.9	0.9	1.0	28.3	20.6
LGP2	1.0	0.7	0.7	1.0	11.5	16.8
TNF-α	1.0	0.6	0.8	1.0	3.1	2.9
IL6	1.0	1.5	1.1	1.0	12.6	18.9

AP treatment

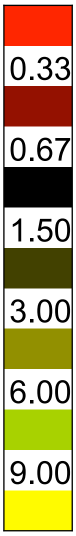

<u>Figure S1.</u> Microarray analysis of mRNAs induced by oligomerized IPS-1 CARD or IPS-1.
HeLa cells stably expressing FK-IPS or FK-IPS CARD were stimulated with AP20187 for the indicated time. Total RNA extracted from these cells was subjected to analysis using a DNA microarray (Genopal, Mitsubishi Rayon) of interferon-stimulated genes and interferon genes. Relative mRNA levels using a control expression as 1.0 are shown.
