# Supplementary material for: Functional Characterization of Domains of IPS-1 Using an Inducible Oligomerization System
Source: PLoS One. 2013 Jan 7;8(1):e53578. doi: 10.1371/journal.pone.0053578 (PMC3538592; doi:10.1371/journal.pone.0053578)
Supplement: Figure S2 — FK-IPS ΔCARDΔTM forms speckle like aggregates in the cytoplasm. HeLa cells stably expressing FK-IPS ΔCARDΔTM were mock treated or treated with AP20187 for 3 h and stained with mitoTracker (mitochondria) and anti-HA antibody. Fluorescent microscopic images of FK-IPSΔCARDΔTM and mitochondria are shown. (PDF) [file pone.0053578.s002.pdf]

Supplementary Figure 2

HeLa FK-IPS ΔCARDΔTM

AP -

AP +

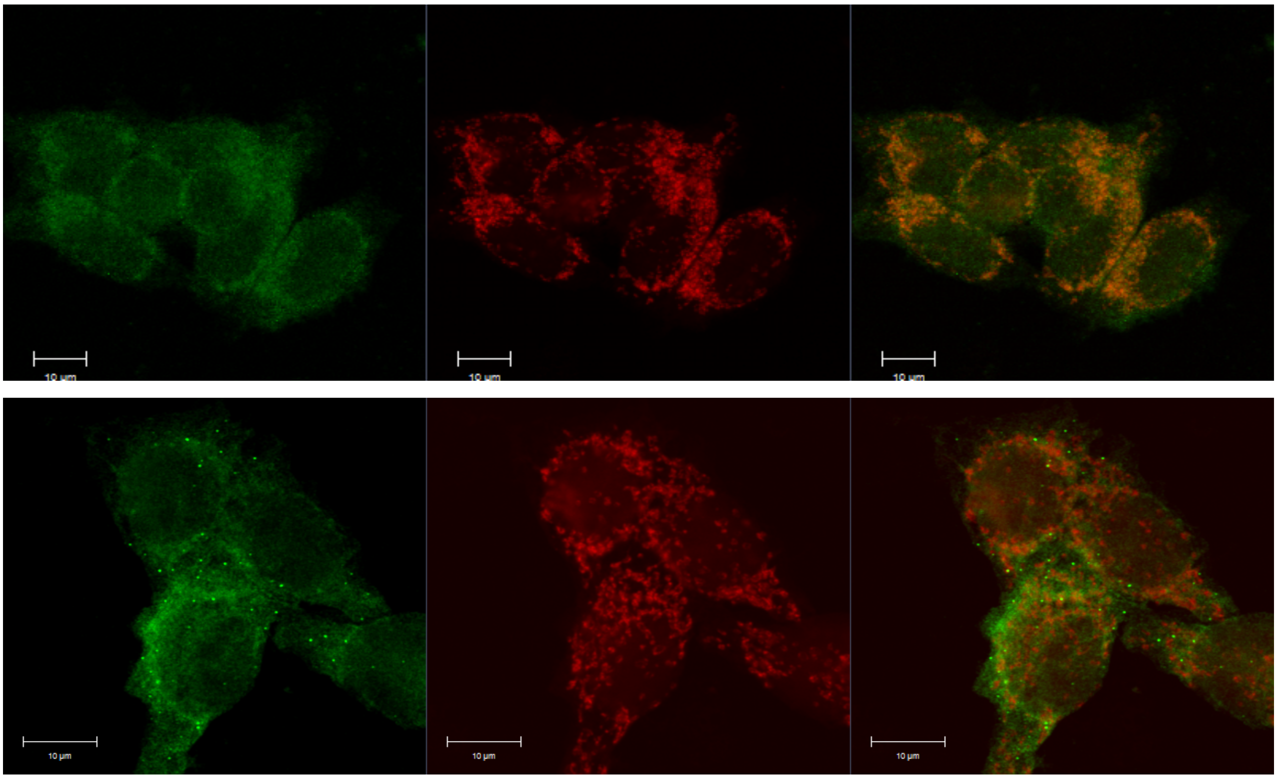

α-HA
(FK-IPS ΔCARDΔTM)

mitochondria

merge

<u>Figure S2.</u> FK-IPS ⊿CARD⊿TM forms speckle like aggregates in the cytoplasm.
HeLa cells stably expressing FK-IPS ⊿CARD⊿TM were mock treated or treated with AP20187 for 3 h and stained with mitoTracker (mitochondria) and anti-HA antibody. Fluorescent microscopic images of FK-IPS⊿CARD⊿TM and mitochondria are shown.
