# Supplementary material for: Functional Characterization of Domains of IPS-1 Using an Inducible Oligomerization System
Source: PLoS One. 2013 Jan 7;8(1):e53578. doi: 10.1371/journal.pone.0053578 (PMC3538592; doi:10.1371/journal.pone.0053578)
Supplement: Figure S3 — MFN1 is dispensable for signaling induced by forced oligomerization of IPS-1. MEFs of MFN1−/− or +/+ were transiently transfected with p-125Luc (reporter for IFN-β promoter activity) together with the indicated FK-IPS fusion constructs. Cells were treated with or without AP20187 for 6 h. Relative luciferase activities were determined as described in Materials and Methods. A representative result of at least two independent experiments is shown. Error bars indicate standard error of triplicate samples. (PDF) [file pone.0053578.s003.pdf]

Supplementary Figure 3

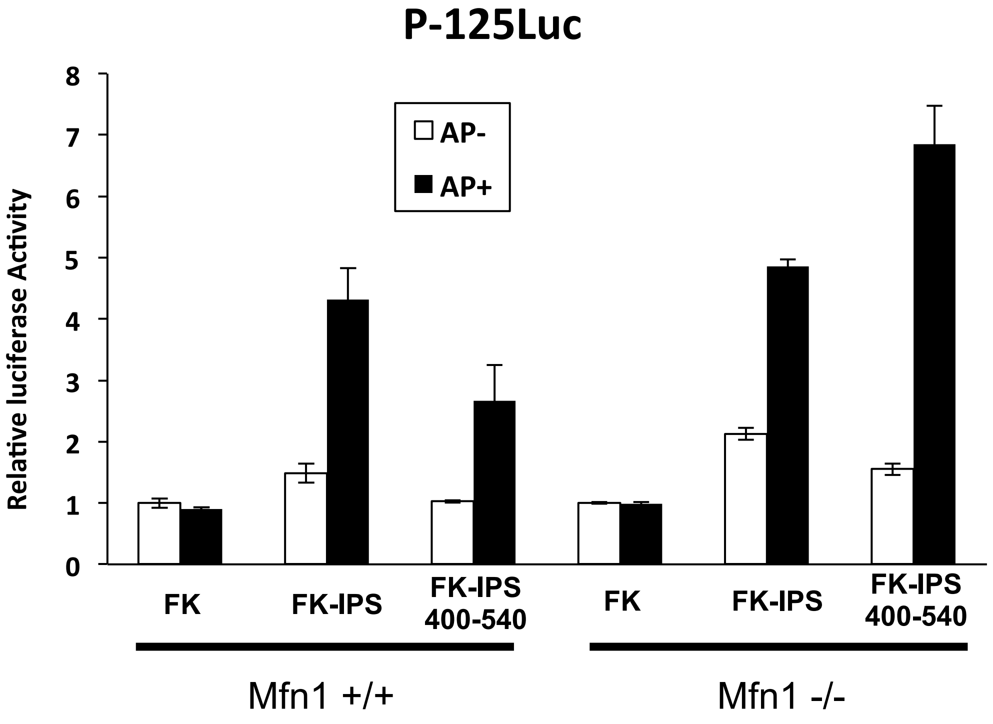

Figure S3. MFN1 is dispensable for signaling induced by forced oligomerization of IPS-1. MEFs of MFN1 -/- or +/+ were transiently transfected with p-125Luc (reporter for IFN-β promoter activity) together with the indicated FK-IPS fusion constructs. Cells were treated with or without AP20187 for 6 h. Relative luciferase activities were determined as described in Materials and Methods. A representative result of at least two independent experiments is shown. Error bars indicate standard error of triplicate samples.
