# Supplementary material for: Functional Characterization of Domains of IPS-1 Using an Inducible Oligomerization System
Source: PLoS One. 2013 Jan 7;8(1):e53578. doi: 10.1371/journal.pone.0053578 (PMC3538592; doi:10.1371/journal.pone.0053578)
Supplement: Figure S4 — FK-IPS 400–508 can activate IRF-responsive promoter upon oligomerization. HEK 293T cells were transiently transfected with p-55C1BLuc together with the FK or FK-IPS 400–540 constructs. Cells were treated with or without AP20187 for 6 h. Relative luciferase activities were determined as described in Materials and Methods. A representative result of at least two independent experiments is shown. Error bars indicate standard error of triplicate samples. (PDF) [file pone.0053578.s004.pdf]

Supplementary Figure 4

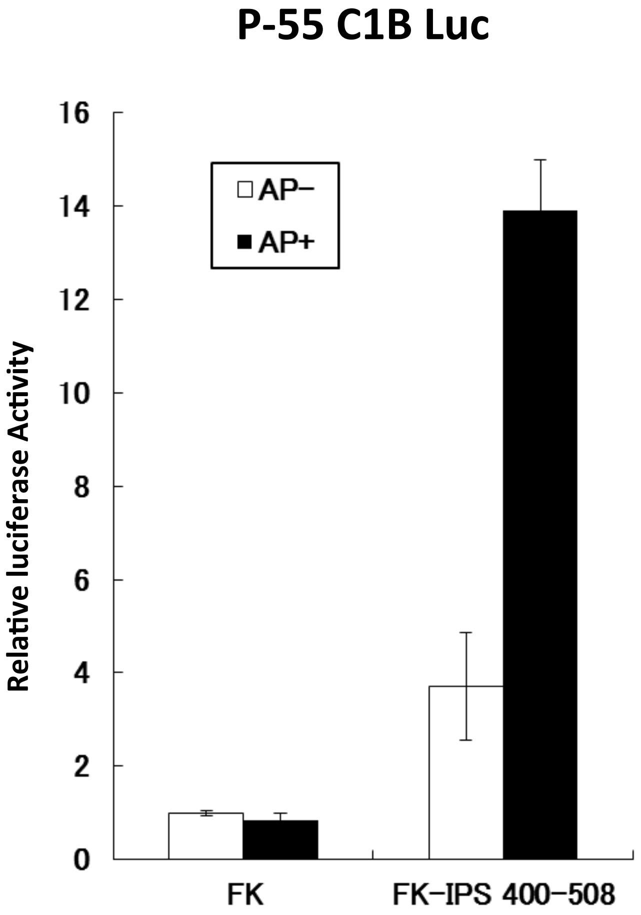

Figure S4. FK-IPS 400-508 can activate IRF-responsive promoter upon oligomerization. HEK 293T cells were transiently transfected with p-55C1BLuc together with the FK or FK-IPS 400-540 constructs. Cells were treated with or without AP20187 for 6 h. Relative luciferase activities were determined as described in Materials and Methods. A representative result of at least two independent experiments is shown. Error bars indicate standard error of triplicate samples.
