# Supplementary material for: Functional Characterization of Domains of IPS-1 Using an Inducible Oligomerization System
Source: PLoS One. 2013 Jan 7;8(1):e53578. doi: 10.1371/journal.pone.0053578 (PMC3538592; doi:10.1371/journal.pone.0053578)
Supplement: Figure S5 — IPS-1Δ100–500 (mini-MAVS) failed to activate signaling in the absence of endogenous IPS-1. IPS-1−/− or +/+ MEFs were transiently transfected with luciferase reporter plasmid, p-55C1BLuc together with IPS-1(MAVS), IPS-1Δ100–500 (mini-MAVS), or control vector. Relative luciferase activities were determined as described in Materials and Methods. A representative result of at least two independent experiments is shown. Error bars indicate standard error of triplicate samples. (PDF) [file pone.0053578.s005.pdf]

Supplementary Figure 5

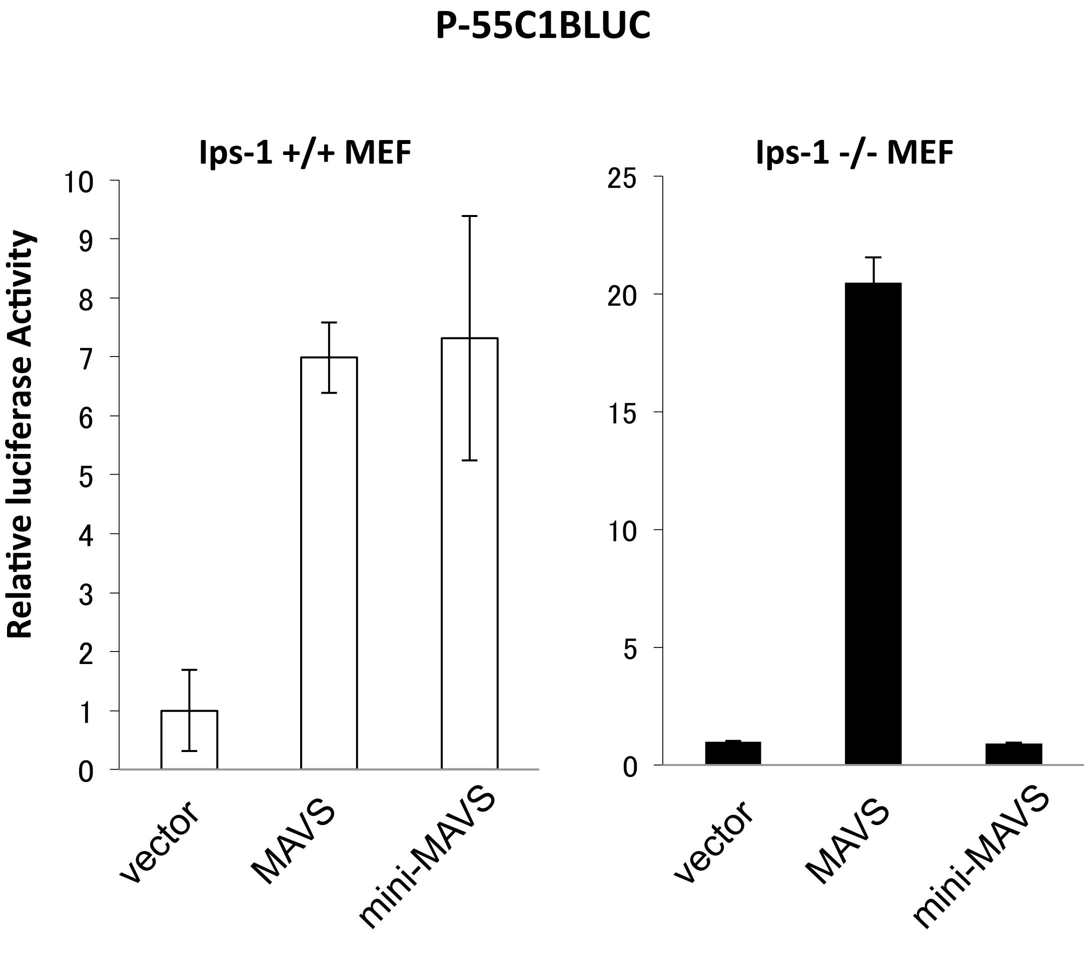

Figure S5. IPS-1⊿100-500 (mini-MAVS) failed to activate signaling in the absence of endogenous IPS-1.

IPS-1 -/- or +/+ MEFs were transiently transfected with luciferase reporter plasmid, p-55C1BLuc together with IPS-1(MAVS), IPS-1D100-500 (mini-MAVS), or control vector. Relative luciferase activities were determined as described in Materials and Methods. A representative result of at least two independent experiments is shown. Error bars indicate standard error of triplicate samples.
