# Supplementary material for: Functional Characterization of Domains of IPS-1 Using an Inducible Oligomerization System
Source: PLoS One. 2013 Jan 7;8(1):e53578. doi: 10.1371/journal.pone.0053578 (PMC3538592; doi:10.1371/journal.pone.0053578)
Supplement: Figure S6 — Recruitment of TRAF6 into NP-40 insoluble fraction upon oligomerization of IPS-1. A. Scheme for isolation of soluble and insoluble fractions by differential centrifugation. B and C. Immunoblot analysis of soluble/insoluble fractions separated by differential centrifugation. FK-IPS ΔCARD stable cells were cultured for 3 h in the absence or presence of AP. Cell lysates were separated by differential centrifugation. FK-IPS ΔCARD and endogenous MFN1, TRAF6, and actin were detected by immunoblotting. (PDF) [file pone.0053578.s006.pdf]

Supplementary Figure 6

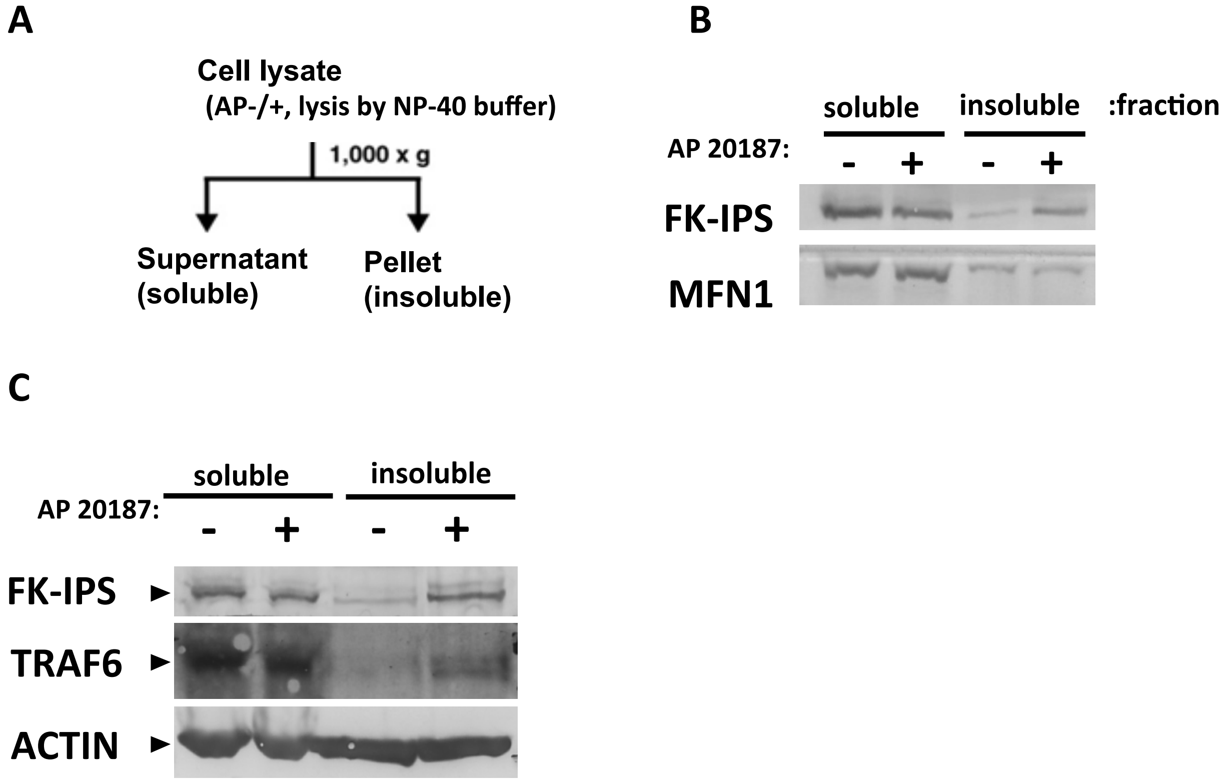

<u>Figure S6.</u> **Recruitment of TRAF6 into NP-40 insoluble fraction upon oligomerization of IPS-1.**

A. Scheme for isolation of soluble and insoluble fractions by differential centrifugation.

B and C. Immunoblot analysis of soluble/insoluble fractions separated by differential centrifugation. FK-IPS DCARD stable cells were cultured for 3h in the absence or presence of AP. Cell lysates were separated by differential centrifugation. FK-IPS DCARD and endogenous MFN1, TRAF6, and actin were detected by immunoblotting.
