# Supplementary material for: Functional Characterization of Domains of IPS-1 Using an Inducible Oligomerization System
Source: PLoS One. 2013 Jan 7;8(1):e53578. doi: 10.1371/journal.pone.0053578 (PMC3538592; doi:10.1371/journal.pone.0053578)
Supplement: Figure S7 — Involvement of CARD9 in NF-κB dependent pathway. A. HeLa FK-IPS#48 cells were transfected with N.C. siRNA or CARD9 targeted siRNA for 48 h, and the knockdown of CARD9 was analyzed by RT-PCR. B, C and D. HeLa FK-IPS#48 cells were transfected with N.C. siRNA or CARD9 targeted siRNA for 48 h, then mock treated or treated with AP20187 for 3 h. Cellular RNA were extracted and analyzed for IFN-β (B), Il-6 (C) or Il-1β (D) mRNA by qPCR. Representative data of at least two independent experiments are shown. Error bars: standard error of triplicated samples. Statistical analyses were conducted with an unpaired t test, with values of p<0.05 considered statistically significant. *p<0.05. (PDF) [file pone.0053578.s007.pdf]

Supplementary Figure 7

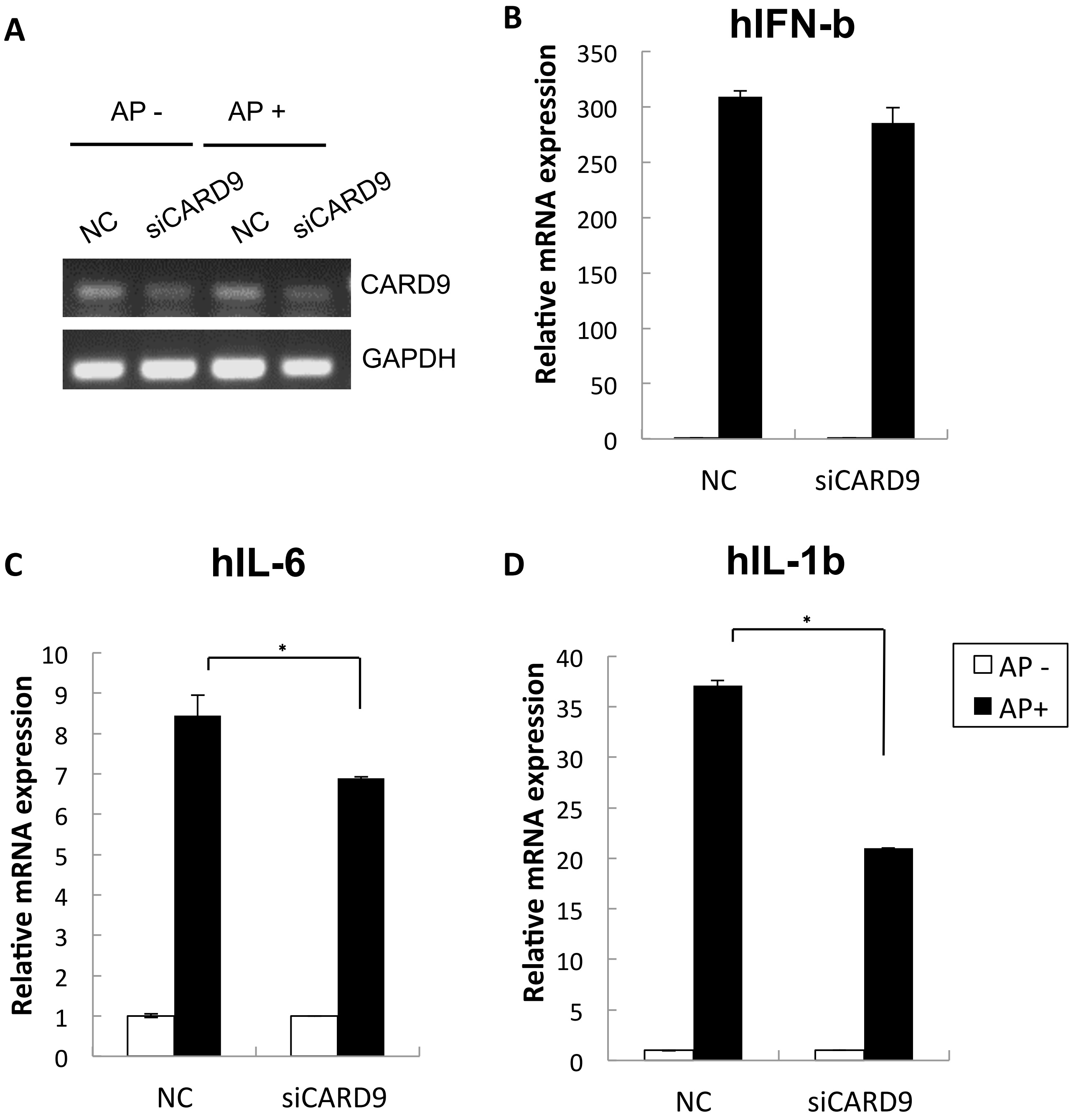

<u>Figure S7.</u> Involvement of CARD9 in NF-kB dependent pathway.
A. HeLa FK-IPS#48 cells were transfected with N.C. siRNA or CARD9 targeted siRNA for 48 h, and the knockdown of CARD9 was analyzed by RT-PCR.
B, C and D. HeLa FK-IPS#48 cells were transfected with N.C. siRNA or CARD9 targeted siRNA for 48 h**,** then mock treated or treated with AP20187 for 3 h. Cellular RNA were extracted and analyzed for IFN-β (B), Il-6 (C) or Il-1β (D) mRNA by qPCR. Representative data of at least two independent experiments are shown. Error bars: standard error of triplicated samples. Statistical analyses were conducted with an unpaired t test, with values of $p < 0.05$ considered statistically significant. *: $p < 0.05$.
